# Deep generative abnormal lesion emphasization validated by nine radiologists and 1000 chest X-rays with lung nodules

**DOI:** 10.1371/journal.pone.0315646

**Published:** 2024-12-12

**Authors:** Shouhei Hanaoka, Yukihiro Nomura, Naoto Hayashi, Issei Sato, Soichiro Miki, Takeharu Yoshikawa, Hisaichi Shibata, Takahiro Nakao, Tomomi Takenaga, Hiroaki Koyama, Shinichi Cho, Noriko Kanemaru, Kotaro Fujimoto, Naoya Sakamoto, Tomoya Nishiyama, Hirotaka Matsuzaki, Nobutake Yamamichi, Osamu Abe

**Affiliations:** 1 Department of Radiology and Diagnostic Radiology and Preventive Medicine, The University of Tokyo Hospital, Bunkyo-ku, Tokyo, Japan; 2 Department of Computational Diagnostic Radiology and Preventive Medicine, The University of Tokyo Hospital, Bunkyo-ku, Tokyo, Japan; 3 Center for Frontier Medical Engineering, Chiba University, Chiba, Japan; 4 Department of Computer Science, Graduate School of Information Science and Technology, The University of Tokyo, Bunkyo-ku, Japan; 5 Toranomon Hospital, Minato-ku, Tokyo, Japan; 6 Kanto Rosai Hospital, Kawasaki City, Kanagawa Prefecture, Japan; 7 Teikyo University Hospital, Itabashi-ku, Tokyo, Japan; 8 Center for Epidemiology and Preventive Medicine, Graduate School of Medicine, Tokyo, Bunkyo-ku, Tokyo, Japan; 9 Department of Respiratory Medicine, Graduate School of Medicine, Tokyo, Bunkyo-ku, Tokyo, Japan; Najran University College of Computer Science and Information Systems, SAUDI ARABIA

## Abstract

A general-purpose method of emphasizing abnormal lesions in chest radiographs, named EGGPALE (Extrapolative, Generative and General-Purpose Abnormal Lesion Emphasizer), is presented. The proposed EGGPALE method is composed of a flow-based generative model and L-infinity-distance-based extrapolation in a latent space. The flow-based model is trained using only normal chest radiographs, and an invertible mapping function from the image space to the latent space is determined. In the latent space, a given unseen image is extrapolated so that the image point moves away from the normal chest X-ray hyperplane. Finally, the moved point is mapped back to the image space and the corresponding emphasized image is created. The proposed method was evaluated by an image interpretation experiment with nine radiologists and 1,000 chest radiographs, of which positive suspected lung cancer cases and negative cases were validated by computed tomography examinations. The sensitivity of EGGPALE-processed images showed +0.0559 average improvement compared with that of the original images, with -0.0192 deterioration of average specificity. The area under the receiver operating characteristic curve of the ensemble of nine radiologists showed a statistically significant improvement. From these results, the feasibility of EGGPALE for enhancing abnormal lesions was validated. Our code is available at https://github.com/utrad-ical/Eggpale.

## Introduction

Chest X-ray examination is a cheap and easily performable medical imaging test and is widely used for the screening of various diseases, including heart failure, lung cancer, pneumonia, and tuberculosis. However, the sensitivity of chest X-rays is less than that of computed tomography (CT) [1, 2], and the X-ray sensitivity among radiologists can vary widely [3–6]. In particular, it is difficult to detect lesions that are small, faint, or superimposed on a bone or the heart, and experience is needed to distinguish real lesions from fake lesions, e.g., superimposed vessels and/or bones.

Many image processing methods that can emphasize lesions in chest X-rays have been presented. One type of image processing method is intensity-based (including contrast stretching [7], histogram equalization (HE) [8], and contrast limited adaptive HE (CLAHE) [9]). Another type of image processing method (feature-based enhancement) emphasizes the lesions themselves, such as by using a finite impulse response filter [10], Laplacian of Gaussian (LoG) filter [11], Hessian-LoG filter [12], or wavelet transform [13]. The third type of method includes rib bone suppression techniques [14–16]. However, to the best of our knowledge, no image processing method that can emphasize abnormal lesions but does not alter the normal structure (bone, vessels, etc.) has been reported.

In recent years, image processing using deep learning has become increasingly popular [17]. Thanks to such new sophisticated techniques, the performance of computer-aided detection (CAD) is improving [3, 18–21]. Nevertheless, typical supervised CAD training requires a huge training dataset, in which each sample must be labeled as normal or abnormal. Manual input of each lesion position/shape is also usually needed. Moreover, a multiple-disease dataset is needed in order to build a database that enables CAD to handle multiple types of lesions. These problems have been partly solved by preparing a huge database that includes several frequent pathologies, such as the Chest-Xray14 dataset [22]. So far, however, few single methods that can handle any type of disease/lesion have been reported.

Theoretically, a model trained only with a huge set of normal cases can also detect abnormal lesions by, for example, an out-of-distribution (OoD) detection method [23]. Such a strategy is also often called one-class classifier or unsupervised anomaly detection problem. These new methodologies may enable us to build a multipurpose CAD application. However, a relatively small number of studies have been conducted so far.

Recently, flow-based generative models, which are one of unsupervised deep learning methods, have emerged. The flow-based generative model is a family of generative models, which includes the variational autoencoder (VAE) [24] and generative adversarial network (GAN) [25, 26]. The advantages of a flow-based model over the VAE and GAN include the invertibility of the mapping function and explicit computability of the probability of each model instance [27]. Although many generative-model-based anomaly detection methods have been reported (denoising autoencoder (AE) [28], adversarial AE [29], AnoGAN [30], Efficient GAN [31], *α*-GAN [32], fast AnoGAN [33]), to the best of our knowledge, only one method that uses flow-based generative models as an anomaly detector in medical images has been reported [34].

In this study, we propose a novel abnormality-enhancing method based on flow-based generative models for chest radiographs. The method uses Glow [27], one of the state-of-the-art flow-based generative models. We choose Glow because of the existence and uniqueness of inversion function which maps a point from the latent space to the image space, unlike VAE and GAN. Owing to existence of unique inversion function, the proposed algorithm can generate a unique anomaly-enhanced image for any input image. Firstly, a large dataset of normal chest radiographs is used to train a Glow model. Then, using the mapping function provided by the model, an inputted unseen chest radiograph is mapped to a certain point in the latent space. The enhancement is performed by moving this point away from the “normal chest X-ray hyperplane,” which is a hyperplane that includes all normal data points in the training. In other words, the image point in the latent space is extrapolated so that its distance from the normal hyperplane increases. We introduce a dedicated *L*^*∞*^-norm-based extrapolation method so that the image change can become more lesion-specific. Finally, by using the invertibility of Glow, the enhanced point is mapped back so that the corresponding enhanced image is generated. We named the proposed method Extrapolative, Generative and General-Purpose Abnormal Lesion Emphasizer, or EGGPALE.

EGGPALE only requires normal chest radiographs in its training phase. It only amplifies abnormal lesions and leaves other normal structures unchanged. This is in strong contrast to previous anomaly enhancement methods, in which the alteration of image contrast/edges, vanishing of the rib cage/spinal column, etc., are inevitable. A potential advantage of this “leave normal as is” property is that a physician only has to check the amplified image and does not have to check the original image, so the workload of physicians will not be doubled. We hypothesized that, using only our amplified radiographs (without checking the original images), radiologists can detect tumorous lung lesions more accurately. To prove this hypothesis, image interpretation experiments involving nine radiologists were performed. Radiographs in which tumorous/nodule-like lesions had been validated by CT images were used in the experiments. We intentionally included chest radiographs with tiny nodules that were not obvious in the radiograph but were revealed by CT to evaluate the increase in human sensitivity when using EGGPALE. Although theoretically our method can enhance various types of anomalies (such as heart enlargement, pneumothorax, and pleural effusion), in this study, we focus on the enhancement of nodule-like lesions.

The contributions of this study are as follows:

- A novel abnormality-enhancing method, EGGPALE, is proposed. Using the invertibility of flow-based models and extrapolation in the latent space, EGGPALE can successfully enhance various pathological lesions.
- Image interpretation experiments involving nine radiologists and 1,000 chest X-ray images demonstrated that EGGPALE can increase the sensitivity of radiologists in detecting nodule-like lesions without significantly increasing the image interpretation time.

To the best of our knowledge, this is the first study in which the sensitivity of physicians to chest X-ray lung nodule-like lesions was improved by abnormality enhancement without changing normal structures or image contrast. Moreover, although a number of previous works have synthesized chest X-rays with GANs [35–37], this is the first study in which chest X-ray images were synthesized by a flow-based algorithm.

## Methods

### A. Background—Glow

Glow [27] is a flow-based generative model that can estimate the probability distribution function (PDF) of given training datasets (i.e., images). Simultaneously, it generates a mapping function that maps an image instance to a point in the learned latent vector field in which the PDF becomes a simple explicit form (e.g., a multidimensional Gaussian distribution). Let the image resolution be *w* × *h*. Suppose that **x** represents an image vector (whose length equals *wh*). Let the true PDF of the given image domain (e.g., normal chest X-ray) **x** be *p*(**x**). Consider the estimation of *p*(**x**) by a certain parametric function *p*_*θ*_(**x**), where *θ* is a parameter set to be optimized. Given the training datasets (i.e., X-ray images) **x**^(*i*)^, *i* = 1,2, …, *N*, this problem is formulated as a log-likelihood maximization problem as follows:

$$\text{minimize}\;L\left(\theta \right)=\frac{1}{N}\overset{N}{\underset{i=1}{\sum }}-\text{log}\;p_{\theta }\left(x^{\left(i\right)}\right).$$ (1)

Again, *p*_*θ*_(**x**) is a parametric function that resembles *p*(**x**). The first main idea of a flow-based algorithm [38, 39] is to introduce an invertible function *f*_*θ*_(**x**) and latent expression **z**, for instance, as follows:

$$z\;\sim \;p\left(z\right)≜N\left(0,\;I\right)$$ (2)

$$x=f_{\theta }^{-1}\left(z\right)$$ (3)

where $N\left(0,\;I\right)$ denotes a *wh*-dimensional multivariate standard Gaussian distribution. Note that the number of dimensions of the latent expression **z** is the same as that of the image **x**.

The second key idea is to divide function *f*_*θ*_ into a chain (composition) of invertible functions such that *f*_*θ*_ = *f*_*K*_ ∘ *f*_*k*−1_ ⋯ ∘ *f*_2_ ∘ *f*_1_. Each function *f*_*i*_, *i* = 1, 2, … *K* is a simple and explicitly invertible parametric function whose parameters are also determined by *θ*. The determinant of the Jacobian matrix of *f*_*i*_ should also be calculated explicitly. Then the relationship between **x** and **z** becomes

$$x\overset{f_{1}}{↔}h_{1}\overset{f_{2}}{↔}h_{2}\cdots h_{K-1}\overset{f_{K}}{↔}z,$$ (4)

where **h**_*i*_ = *f*_*i*_(**h**_*i*−1_). This sequence of invertible functions is called a “normalizing flow”. Then, *p*_*θ*_(**x**) can be calculated as follows using the chain rule of composite functions:

$$\begin{array}{ll} \text{log}\;p_{\theta }\left(x\right) & =\text{log}\;p\left(z\right)+\text{log}\left|\text{det}\frac{dz}{dx}\right|\\ & =\text{log}\;p\left(z\right)+\overset{K}{\underset{i=1}{\sum }}\;\text{log}\left|\text{det}\frac{dh_{i}}{dh_{i-1}}\right|, \end{array}$$ (5)

where **h**_0_ ≜ **x** and **h**_*K*_ ≜ **z**. The matrix *d***h**_*i*_/*d***h**_*i*−1_ is the Jacobian matrix of the function *f*_*i*_. Note that $\text{log}\;p\left(z\right)=-\frac{1}{2}wh\;\text{log}\left(2\pi \right)-\frac{1}{2}{\left|z\right|}^{2}$ can be calculated easily because of Eq (2). Using Eq (5), the parameter *θ* of the estimated PDF *p*_*θ*_(**x**) can be estimated by searching for the value of *θ* that minimizes Eq (1).

**Fig 1:**
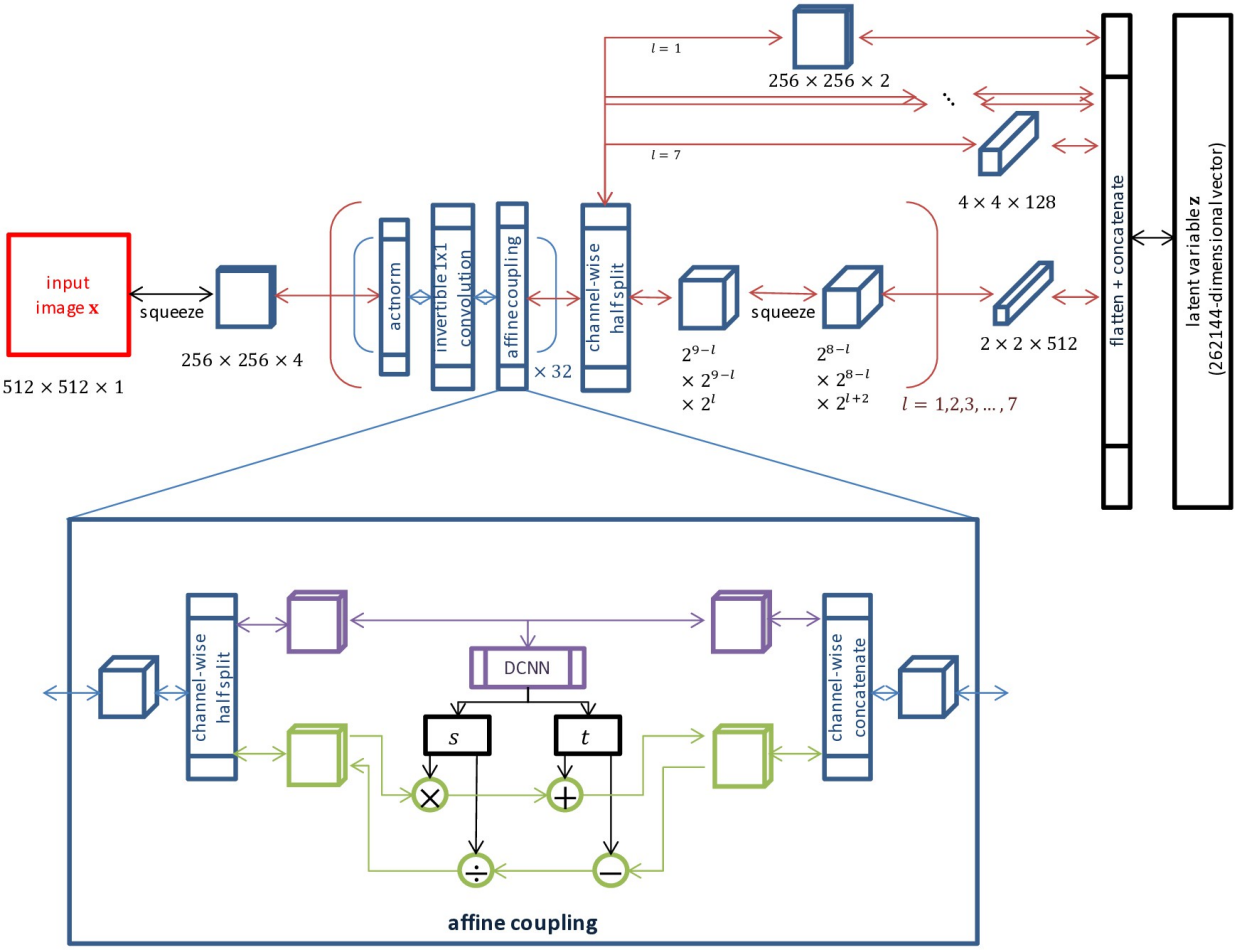
Diagram of the Glow model used.

For conciseness, the details of the Glow framework used are omitted in this paper. A diagram of the entire Glow network used in this study is shown in Fig 1. As described in the original paper on Glow [27], we used actnorm, an invertible 1 × 1 convolution, and affine coupling layers as the components of each step. The number of steps per level was 32, and the total number of levels was seven. Between two adjacent levels, squeeze and split operations were inserted. The image resolution of *w* = *h* = 512 was used. Therefore, both the input **x** and the latent expression **z** were 262,144-dimensional vectors. Most hyperparameters are the same as [27]: the filter (channel) numbers were not changed (please see Fig 1), the input window size is doubled (512×512), the number of epochs was fixed to 22 (we chose a number as large as possible with our computational resource). The other parameters are:

- Number of levels in the Glow model: 7
- Number of affine coupling layers per level: 32
- Number of convolution layers in each affine coupling layer: 3
- Kernel size in each convolutional layer: 3×3

### B. Modification of Glow

We slightly modified the original Glow algorithm. First, the weight matrix **W** of each invertible 1 × 1 convolution (see Fig 1) can collapse in the training phase so that **W** becomes rank deficient and there is no inverse matrix **W**^−1^. This phenomenon sometimes occurred and prevented the invertibility of *f*_*θ*_. To avoid this, at the end of each epoch, **W** was modified as follows using singular value decomposition:

$$\begin{array}{c} W=U\Sigma V^{T}\\ \Sigma_{new}=\text{crop}\left(\Sigma ;\left[10^{-3},\;10^{3}\right]\right)\\ W_{new}=U\Sigma_{new}V^{T}, \end{array}$$ (6)

where the operation crop means the element-wise cropping of the range of values so that all diagonal elements of the diagonal matrix **Σ**_*new*_ are in the range of [10^−3^, 10^3^]. Note that this operation ensures the existence of (**W**_*new*_)^−1^ = **V**(**Σ**_*new*_)^−1^**U**^*T*^.

The second modification is that the distribution of *p*(**z**) is fixed to the standard Gaussian distribution $N\left(0,\;I\right)$ instead of a parametrized (non-standard) Gaussian distribution. This is because we need the PDF of **z** to be isotropic so that the Euclidean and *L*^*∞*^ distances in the latent space have a sense.

### C. Normal chest X-ray hyperplane

**Fig 2:**
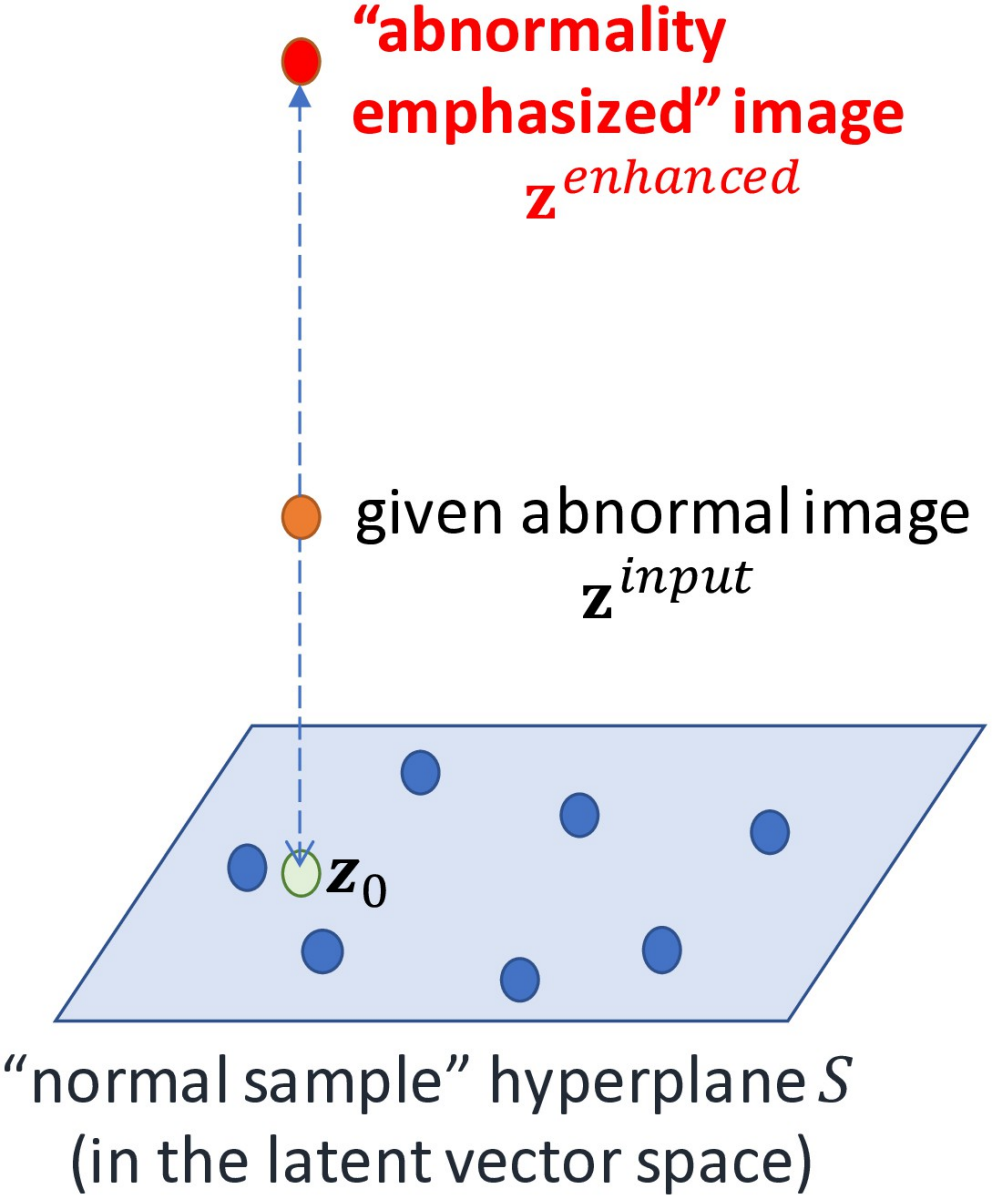
Normal sample hyperplane *S*.*z*_0_ is a projected point.

The input image **x** is first mapped to a point **z** in the latent space using **z** = *f*_*θ*_(**x**), and then the anomaly is amplified in the latent space as follows. The simplest way is to move z away from the origin **O**, which corresponds to the trained average image, in the latent space. However, this method often results in significant deformation of the original image, such as distortions in the thoracic cage. To address this issue, our study adopts a more sophisticated approach utilizing the normal chest X-ray hyperplane *S* (refer to Fig 2).

Let **x**^(*i*)^, *i* = 1, 2, …, *N* be the training datasets (normal cases only). Suppose that *N* < *wh*. Let the mapped points of **x**^(*i*)^ be **z**^(*i*)^ = *f*_*θ*_ (**x**^(*i*)^). Then, all these *N* points in the latent space, **z**^(*i*)^, *i* = 1, 2, …, *N*, as well as the origin **O**, span an *N*-dimensional hyperplane. We call this hyperplane the normal chest X-ray hyperplane *S*.

Suppose that the input image (for abnormality enhancement) is **x**^*input*^. Let the corresponding point in the latent space be **z**^*input*^ = *f*_*θ*_(**x**^*input*^). Then, consider the perpendicular line from **z**^*input*^ down to hyperplane *S*. Let the intersection of this perpendicular line and *S* be ***z***_0_ (see Fig 2). Note that the corresponding image $x_{0}=f_{\theta }^{-1}\left(z_{0}\right)$ can be regarded as a “virtually normalized” image of the given (possibly abnormal) image **x**^*input*^. How to calculate *S* and ***z***_0_ is described in S1 File.

Then, for example, a simple extrapolation (with Euclidean distance) can be performed as

$$z^{enhanced}=\gamma \cdot z^{input}+\left(1-\gamma \right)\cdot z_{0}.$$ (7)

The parameter *γ* determines the strength of abnormality enhancement. When 1 < *γ*, the corresponding abnormality-enhanced image $x^{enhanced}=f_{\theta }^{-1}\left(z^{enhanced}\right)$ can be created.

### D. Extrapolation with *L*^*∞*^ distance

Although the Euclidean distance-based extrapolation works well, it tends to excessively amplify the shift, rotation, local deformation, and so forth, as well as abnormal lesions. We hypothesize that this phenomenon is owing to the fact that, in Eq (7), all elements of **z** are altered simultaneously. Instead, we wish to amplify only certain elements of **z** that are responsible for the abnormal lesions, leaving the other elements as they are. To solve this problem, we introduce an *L*^*∞*^ distance-based extrapolation.

**Fig 3:**
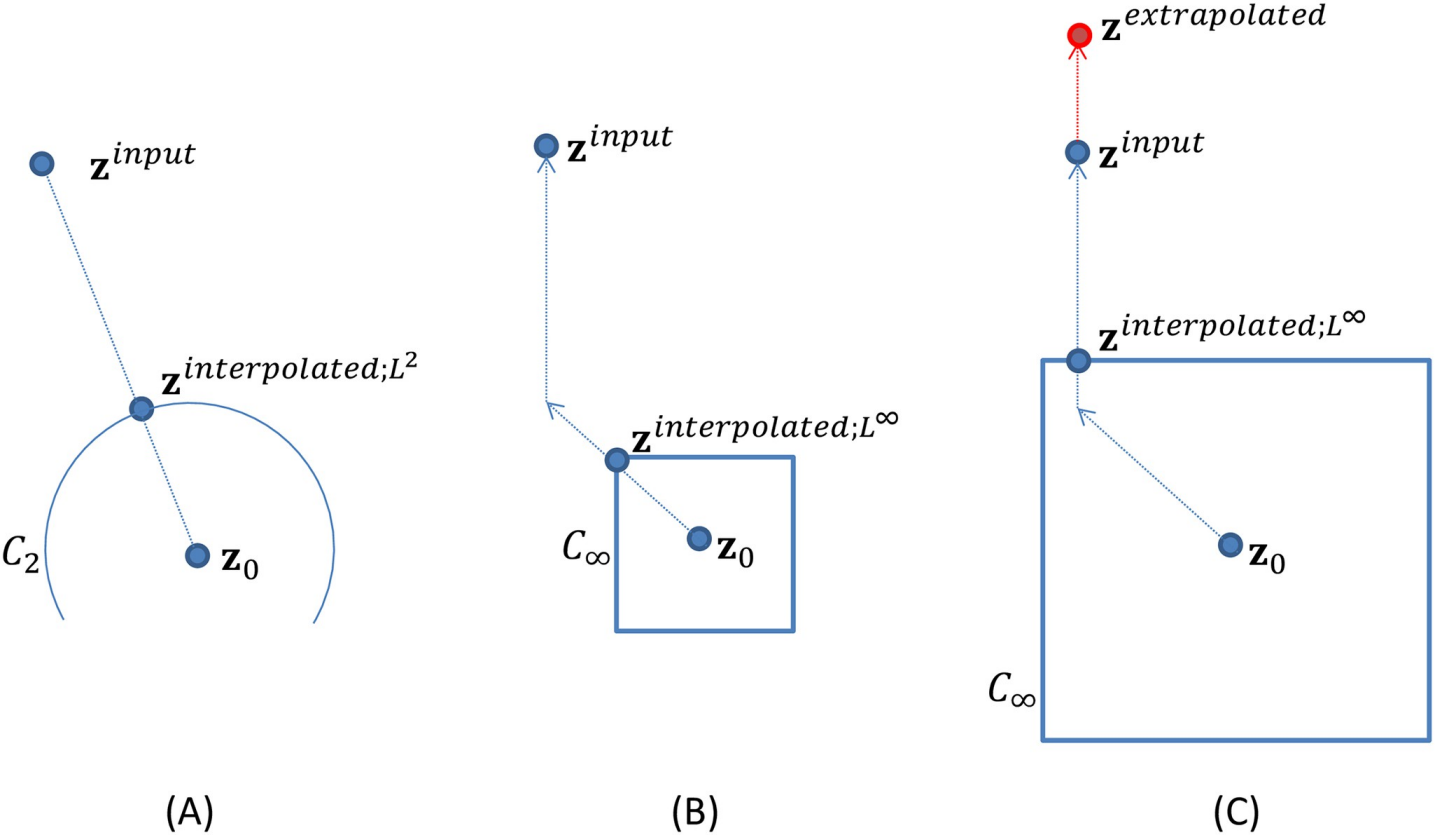
(A) Interpolation with *L*^2^ norm, and interpolation with *L*^*∞*^ norm when *γ* is (B) small and (C) large.

Fig 3 shows an outline of *L*^*∞*^ distance-based extrapolation. This extrapolation consists of three steps: (1) finding **z**_0_, (2) interpolation using the *L*^*∞*^ distance, and (3) final extrapolation with the Euclidean distance. Operation (1) is the same as that in the previous section and the projected point **z**_0_ is determined on *S*. Then, in operation (2), interpolation between **z**^*input*^ and **z**_0_ is performed. Let the interpolated point between **z**^*input*^ and **z**_0_ be $z^{interpolated;L^{2}}$. For the sake of explanation, the interpolation operation can be rewritten in a Euclidean distance-based manner:

$$z^{interpolated;L^{2}}=\text{arg}\;\underset{z\in C_{2}}{\text{min}}\;∥z-z^{input}{∥}_{2}$$ (8)

for hypersphere *C*_2_ = {**z**│‖**z** − **z**_0_‖_2_ ≤ *γ* ⋅ ‖**z**^*input*^ − **z**_0_‖_2_} (Fig 3A). This definition is equivalent to Eq (7) when 0 ≤ *γ* ≤ 1. Then, we replace the *L*^2^ norm with the *L*^*∞*^ and *L*^1^ norms, as follows:

$$z^{interpolated;L^{\infty }}=\text{arg}\;\underset{z\in C_{\infty }}{\text{min}}∥z-z^{input}{∥}_{1},$$ (9)

for hypercube *C*_∝_ = {**z**│‖**z** − **z**_0_‖_∝_ ≤ *γ* ⋅ ‖**z**^*input*^ − **z**_0_‖_∝_} (Fig 3B and 3C). Intuitively, this means that the point $z^{interpolated;L^{\infty }}$ moves along a polygonal line, instead of a straight line, between **z**_0_ and **z**^*input*^. When *γ* increases from 0 to 1, the point $z^{interpolated;L^{\infty }}$ first moves from **z**_0_ by an *L*^*∞*^ or chessboard distance (i.e., diagonally), and then moves by an *L*^1^ or Manhattan distance (i.e., horizontally or vertically) toward **z**^*input*^. The actual calculation can simply be performed by the following element-wise range-cropping function [40]:

$$\begin{array}{l} {\left\{z^{interpolated;L^{\infty }}\right\}}_{j}\\ =\text{crop}\left({\left\{z^{input}\right\}}_{j};\;\left[{\left\{z_{0}\right\}}_{j}-\Gamma ,{\left\{z_{0}\right\}}_{j}+\Gamma \right]\right)\\ =\left\{\begin{array}{cc} {\left\{z_{0}\right\}}_{j}-\Gamma & \text{if}\;{\left\{z^{input}\right\}}_{j}<{\left\{z_{0}\right\}}_{j}-\Gamma \\ {\left\{z^{input}\right\}}_{j} & \text{if}\;{\left\{z_{0}\right\}}_{j}-\Gamma \leq \;{\left\{z^{input}\right\}}_{j}\leq {\left\{z_{0}\right\}}_{j}+\Gamma \\ {\left\{z_{0}\right\}}_{j}+\Gamma & \text{if}\;{\left\{z_{0}\right\}}_{j}+\Gamma <{\left\{z^{input}\right\}}_{j} \end{array},\right. \end{array}$$ (10)

where {⋅}_*j*_ denotes the *j*th element and and *Γ* = *γ* ⋅ ‖**z**^*input*^ − **z**_0_‖_∞_. As a result, many elements of $z^{interpolated;L^{\infty }}$ are the same as those of **z**^*input*^. Then, (3) the final extrapolation is performed between **z**^*input*^ and $z^{interpolated;L^{\infty }}$ using a Euclidean distance,

$$z^{extrapolated}=\beta \cdot z^{input}+\left(1-\beta \right)\cdot z^{interpolated;L^{\infty }},$$ (11)

with the extrapolation parameter 1 < *β*. Because many elements of $z^{interpolated;L^{\infty }}$ are the same as those of **z**^*input*^, this extrapolation works for only a small number of elements, so only abnormal lesions are enhanced. In this study, the parameters *γ* = 0.2 and *β* = 1.2 were used, which were determined experimentally. (Grid search with subjective image evaluation by a radiologist was performed to determine optimal parameter values. Too large value of *β* can lead to large local or global deformation of the resulting image.) Finally, the enhanced image $x^{extrapolated}=f_{\theta }^{-1}\left(z^{extrapolated}\right)$ is generated using Glow.

**Fig 4:**
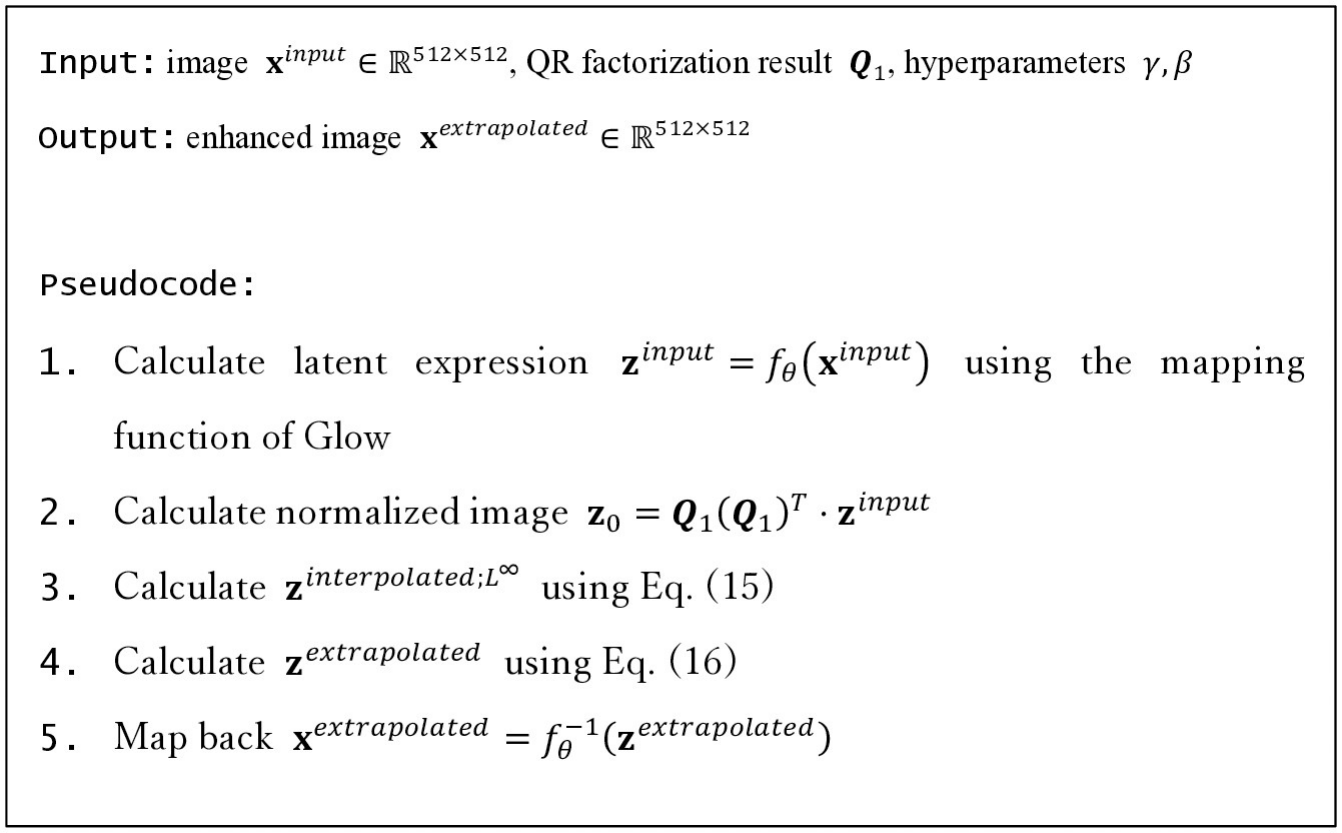
Pseudocode of the proposed EGGPALE.

Fig 4 is a pseudocode of the proposed EGGPALE enhancement.

### E. Experimental settings

This retrospective study was approved by our institutional review board (the University of Tokyo Hospital, IRB protocol number 2561-(19), date of approval 2020/9/16). Informed consent was waived in this retrospective study (which was approved by the IRB). The data were accessed for research on March 16th, 2021. The authors did not have access to information that could identify individual participants during or after data collection. We used the ChestX-ray14 open dataset [22] for the training in the proposed method. Among the 112,120 chest radiographs in ChestX-ray14, we extracted 39,302 radiographs that are labeled as normal and whose radiation direction is posterioanterior (PA). Then we randomly selected approximately 70% of them, resulting as 27,504 normal chest radiographs (*N* = 27,504). Some other radiographs with abnormal labels were also used in our subjective and qualitative experiment.

**Fig 5:**
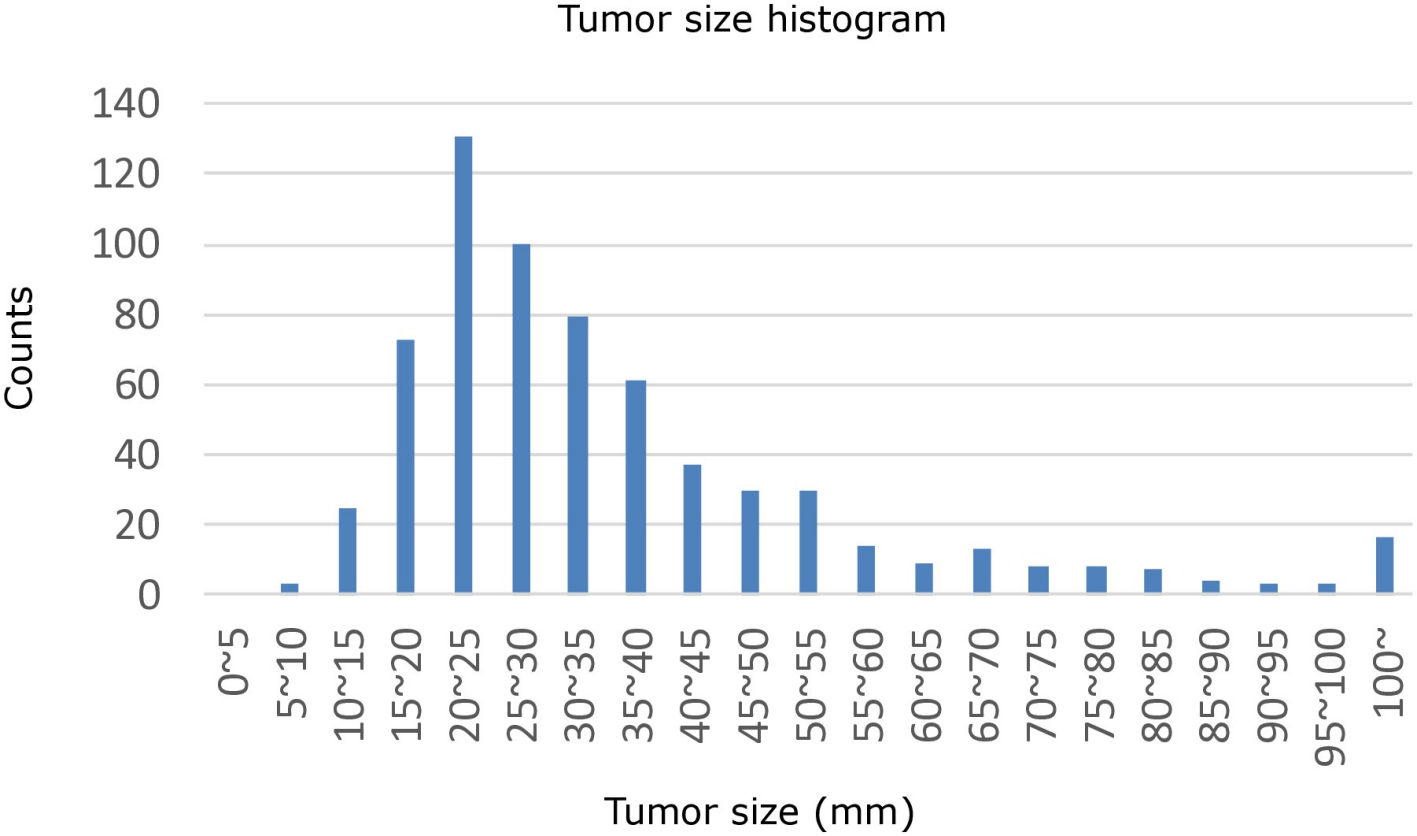
Histogram of tumorous lesion sizes.

For our quantitative blind image-reading experiment, we utilized our domestic radiograph dataset, which was validated by CT. Positive and negative cases were collected from the University of Tokyo Hospital. For the positive cases, the inclusion criteria are (1) chest CT examinations performed in the University of Tokyo Hospital from January 2017 to June 2018 and (2) their radiological diagnosis reports include “lung cancer” or “suspected lung cancer.” A total of 604 cases met these criteria, although several cases were excluded because no corresponding chest radiograph was available (one month before–one month after the CT examination). Cases with diseases (pneumonia, pleural effusion, etc.) unrelated to lung field/hilar/mediastinal masses/nodules were also excluded. After exclusion, 509 cases met the criteria. We used 100 of the 509 cases in the development and the subjective qualitative study, and the other 409 cases in the quantitative image-reading experiment. Note that we did not set any tumor-size-based criterion. Fig 5 shows a histogram of the sizes of the tumorous lesions included in our positive cases (measured in CT images).

Negative cases were collected from our health check program in the University of Tokyo Hospital. The inclusion criteria are (1) chest CT examinations performed for the health check program, (2) negative findings in chest CT reports, and (3) the corresponding chest radiographs were available (one month before–one month after the CT examination). Finally, 591 cases were selected randomly from the cases meeting the criteria.

Before both the training and the experiments, all images were preprocessed as follows. Firstly, the pixel intensities were cropped so that the range of intensity was [0,255]. In range cropping, we used the DICOM tags “window center / window width” (tag IDs: (0028,1050) and (0028,1051), respectively) and the indicated range was then rescaled to [0,255]. Then, the images were resized to a width of 1,024 pixels per image. If necessary, the top/bottom borders were also truncated so that the image height was also 1,024 pixels. Before processing by Glow, images were down-sampled to 512×512 pixels. On the other hand, after enhanced image generation, images were up-sampled to 1024×1024 pixels (using bicubic interpolation). Therefore, all image interpretation experiments were performed by radiologists using images with 1024×1024 pixels.

Glow was trained using 27,504 chest radiographs from the ChestX-ray14 dataset as described above. For the training, a Reedbush-L supercomputer system was used. We used one node with two Intel Xeon E5-2695v4 processors, a memory of 256 GB, and four GPUs (Tesla P100, NVIDIA Corporation, Santa Clara, CA). Data parallelism using Horovod and TensorFlow was utilized. The minibatch size was four. No early stopping was used, and the training duration was 168 h (the upper limit of the supercomputer system usage). The loss curve in the training phase is shown in the S2 File.

Abnormality enhancement was performed using the trained Glow system. The normal chest X-ray hyperplane was also calculated using all 27,504 training cases. The QR factorization of **Z**^*training*^ (whose size was 262,144 × 27,504) was also performed by Reedbush-L using the ScaLAPACK library [41]. In the abnormality enhancement, we did not have to use data parallelism, and thus only one GPU was used for enhancing images. Processing of one radiograph took approximately 5 s.

After processing to enhance the abnormality 1,000 radiographs (409 positives and 591 negatives, as described above), a quantitative blind image-reading experiment was performed. Nine radiologists with 3, 3, 3, 4, 5, 7, 9, 18 and 31 years of experience were assigned to this study. The 1,000 radiographs were divided into two groups, A and B. Each radiologist was first asked to interpret a shuffled mixture of original radiographs of A and enhanced radiographs of B. Then, two weeks later, each radiologist was asked to interpret enhanced radiographs of A and original radiographs of B. The interpretation was carried out using our domestically developed browser-based software. Each radiologist inputted the existence or non-existence of nodulous lesion(s) for each lung (including the adjacent mediastinal area) for each case. Thus, a total of 1,000 cases × 2 enhanced/unenhanced × 2 lung fields = 4,000 inputs was evaluated by each radiologist. The reaction time of each interpretation was also collected. After all images were interpreted by the nine radiologists, each input was judged as correct or incorrect on the basis of ground truth information determined using CT images. The accuracy, sensitivity, and specificity were calculated for each radiologist with and without the proposed abnormality enhancement. Furthermore, the receiver operating characteristic (ROC) of the ensemble of all nine radiologists was also analyzed. The statistical analysis was performed using R 4.0.2. (The R Project for Statistical Computing, Vienna, Austria).

As a baseline method, we also performed an experiment with an open-source rib bone suppression method [42]. Apart from the different enhancing/suppressing methods, the experimental setting was the same as that of the main experiment described above.

Finally, we performed an experiment in which the amount of nodulous region enhancement was quantitatively evaluated. First, we manually inputted the region of interest (ROI) of each nodulous lesion in each of 100 cases (the dataset for the subjective qualitative study). This dataset included 133 nodules. We also semiautomatically extracted the lung field of each of the 100 images. Then, the contrast-to-noise ratio (CNR) was calculated for each nodule as follows:

$$CNR_{-}=\frac{\mu_{nodule}-\mu_{lungfield}}{\sigma_{lungfield}},$$ (12)

where *μ*_*nodule*_, *μ*_*lungfield*_, and *σ*_*lungfield*_ are the mean intensity of the nodule ROI, the mean intensity of the lung field, and the standard deviation of the lung field, respectively. In the same way, the CNR of each EGGPALE-enhanced image, or *CNR*_+_, was also calculated. Then, the relative improvement of CNR by EGGPALE was estimated as follows:

$$\Delta_{CNR}=CNR_{+}-CNR_{-}$$ (13)

The distribution of Δ_*CNR*_ was evaluated by plotting a histogram.

## Results

**Fig 6:**
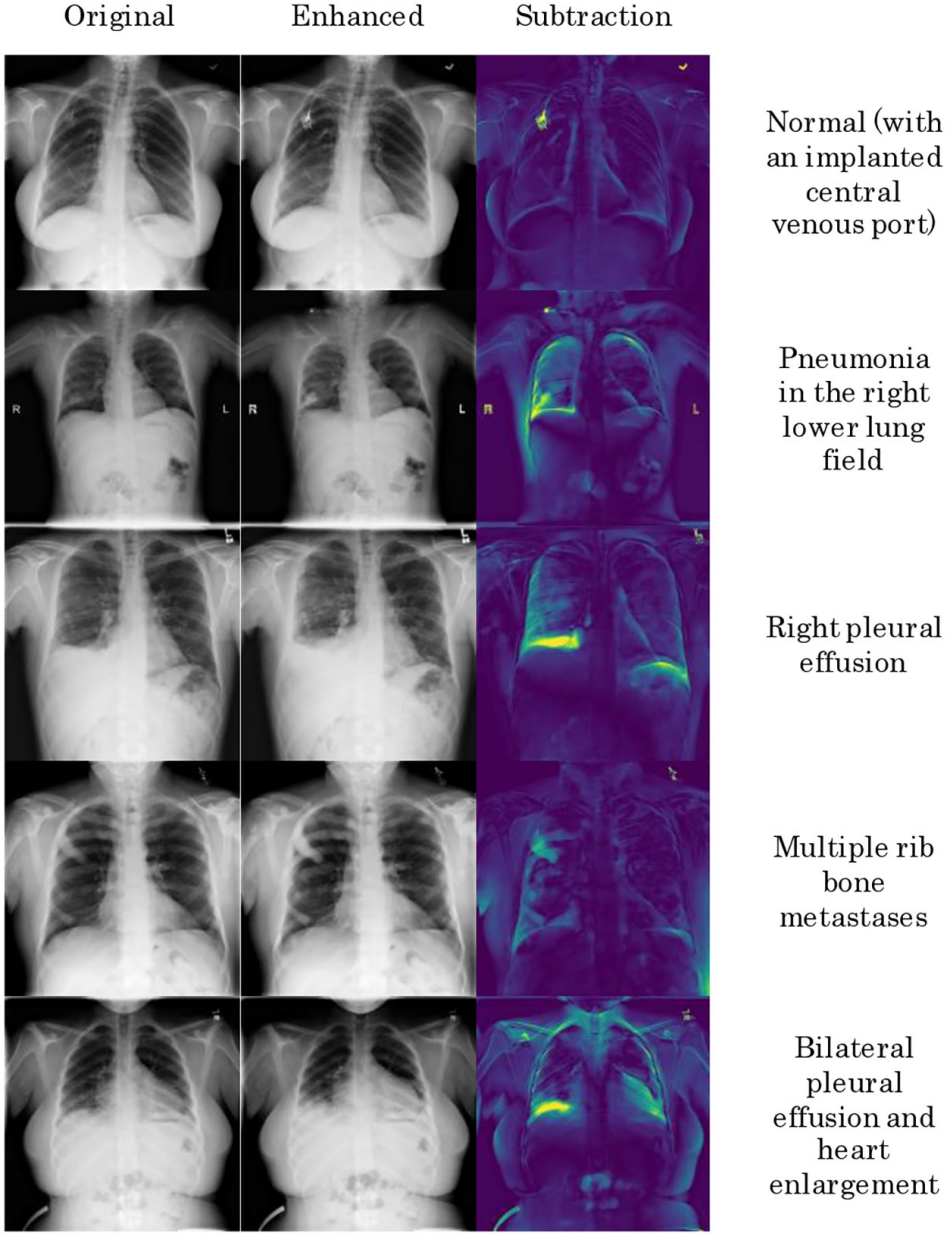
Qualitative results of several types of abnormalities.

**Fig 7:**
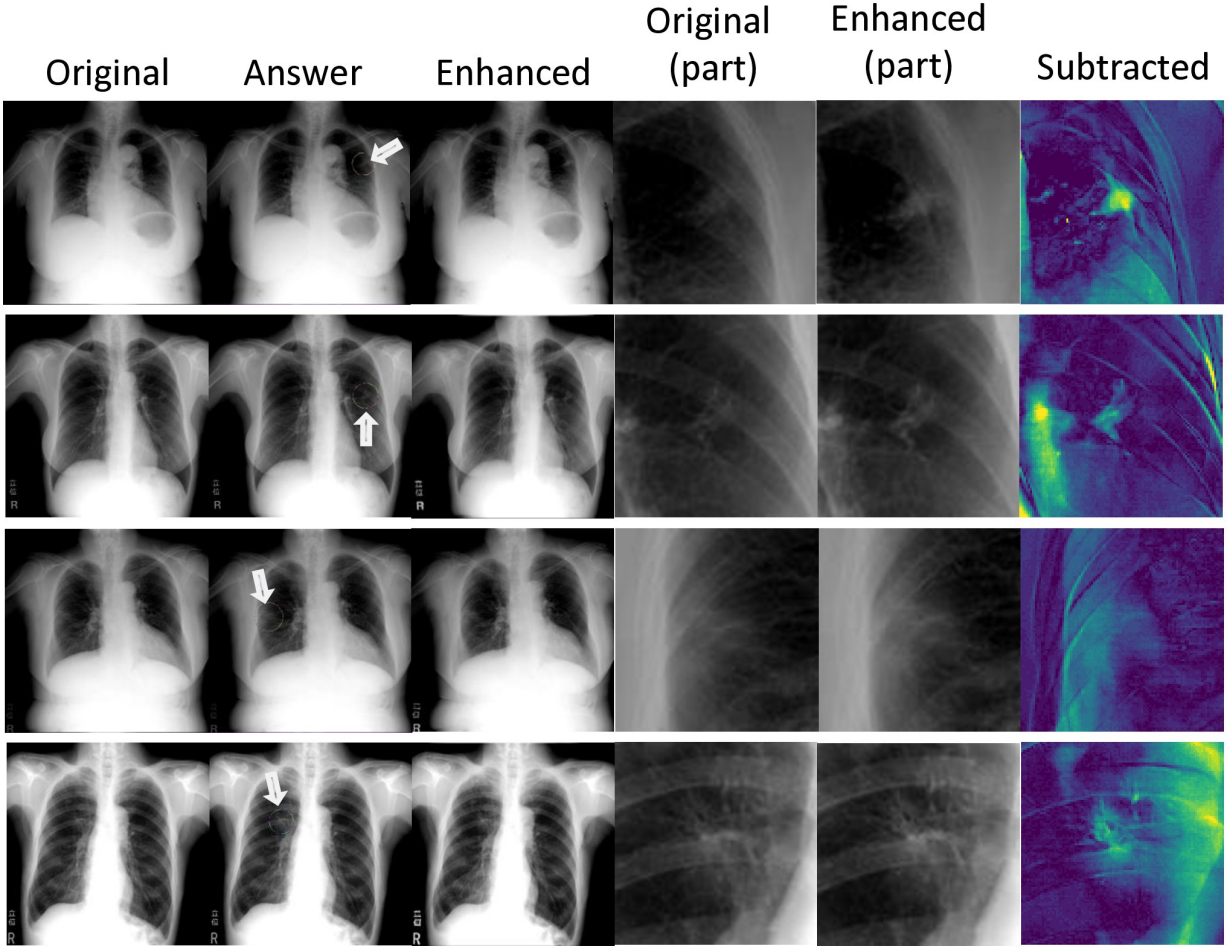
Qualitative results of four cases of faint nodules. The effect of enhancement is shown in the subtracted images (right column). The answer column indicates the true nodule that should be detected by radiologists. The true lesions are indicated by arrows.

Our proposed EGGPALE method successfully enhanced all inputted images. Examples of normal cases and cases with nodular lesions, pneumonia/consolidation, pleural effusion, heart enlargement, etc. are shown in Figs 6 and 7. As shown, local abnormal structures were successfully enhanced with apparently no change to normal structures.

To confirm that EGGPALE does not largely change normal chest X-ray images, the Turing test was performed using 100 normal images. Two radiologists independently evaluated 100 pairs of images, each containing one with EGGPALE processing and one without. The radiologists were tasked with determining whether each image was processed with EGGPALE or not. In total, 200 images were shuffled and presented to each radiologist for judgment. The resulting accuracies were 0.79 and 0.63 for the two radiologists, indicating that 21–37% of images were misclassified (either original images misclassified as EGGPALE-processed or vice versa). Based on these findings, we conclude that our proposed EGGPALE algorithm minimally impacts image quality, as misjudgments occurred within an acceptable range.

**Fig 8:**
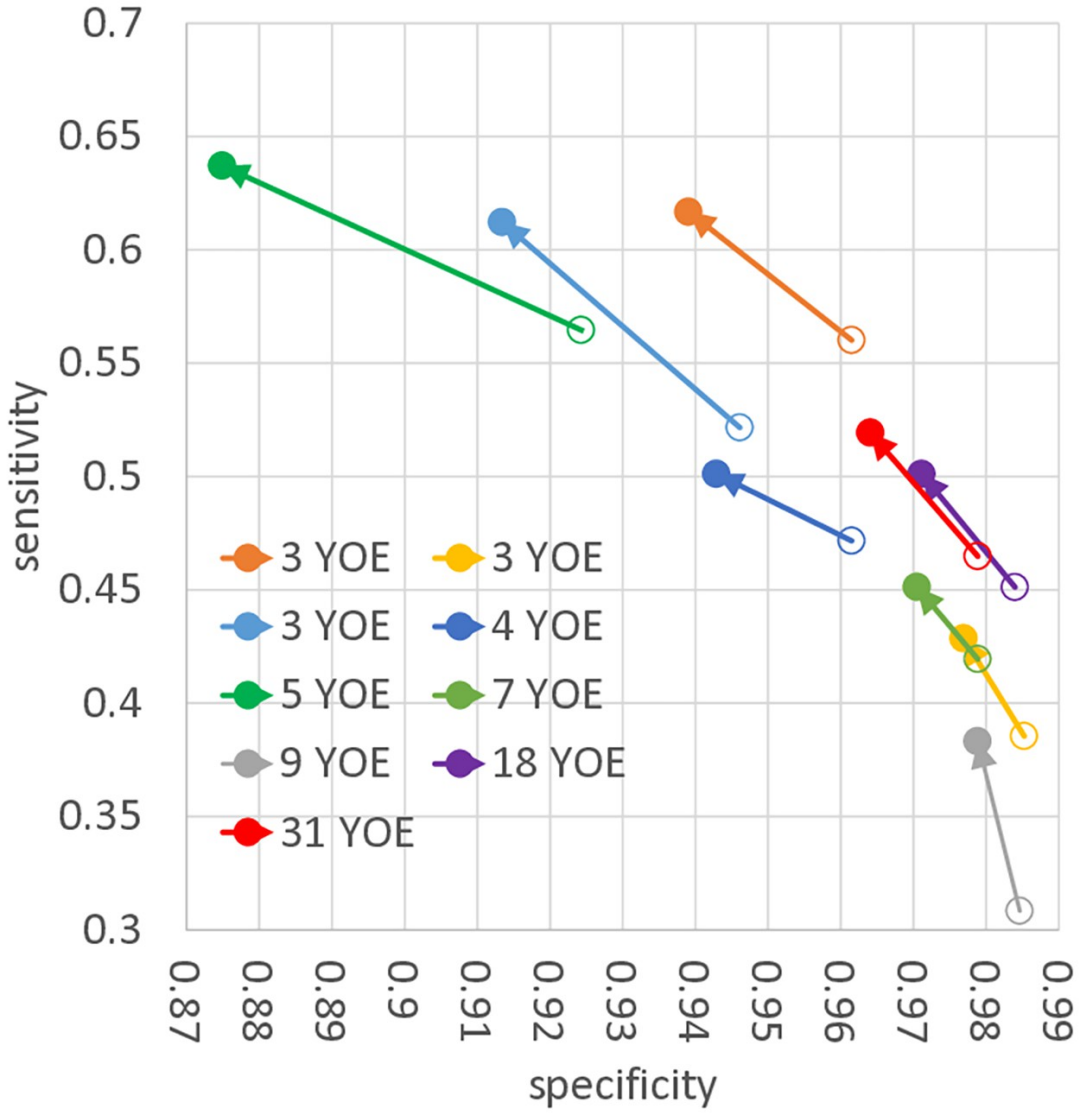
Changes in sensitivity and specificity of each radiologist with and without EGGPALE enhancement. Open and closed circles represent results without and with EGGPALE, respectively. YOE = years of experience.

The results of the quantitative blind image-reading experiment are shown in Table 1 and Figs 7 and 8. Fig 7 demonstrates the results of nodular lesion emphasis. In Table 1 and Fig 8, the changes in the sensitivity and specificity of each radiologist by EGGPALE are shown. The average improvement of sensitivity was 0.0559, whereas the average decrease in specificity was 0.0192. Paired Student’s t-tests showed significant differences for both (*p* = 1.79 × 10^−5^ and *p* = 0.0017, respectively). The sensitivity improved and the specificity decreased for all nine radiologists. Therefore, we concluded that EGGPALE successfully increased the sensitivity with little deterioration of specificity. On the other hand, the sensitivity deteriorated for all nine radiologists when using the bone suppression method [42]. Therefore, the superiority of the proposed model compared with the existing model was validated.

**Table 1:** Sensitivities, specificities, and accuracies of nine radiologists with and without EGGPALE enhancement. Each row represents each radiologist. Each column represents whether the images shown were emphasized by EGGPALE or not.

years of experience	original images			EGGPALE			(EGGPALE—original)			bone suppression			(bone supp.- original)		
	sens.	spec.	accu.	sens.	spec.	accu.	sens.	spec.	accu.	sens.	spec.	accu.	sens.	spec.	accu.
3 YOE	0.5601	0.9615	0.8730	0.6168	0.9390	0.8679	0.0567	-0.0225	-0.0051	0.4966	0.9583	0.8565	-0.0635	-0.0032	-0.0165
3 YOE	0.3855	0.9852	0.8530	0.4286	0.9769	0.8559	0.0431	-0.0084	0.0029	0.3243	0.9897	0.8430	-0.0612	0.0045	-0.0100
3 YOE	0.5215	0.9461	0.8525	0.6122	0.9133	0.8468	0.0907	-0.0328	-0.0057	0.4580	0.9230	0.8205	-0.0635	-0.0231	-0.0320
4 YOE	0.4717	0.9615	0.8535	0.5011	0.9428	0.8453	0.0295	-0.0187	-0.0082	0.2857	0.9865	0.8320	-0.1859	0.0250	-0.0215
5 YOE	0.5646	0.9243	0.8450	0.6372	0.8748	0.8223	0.0726	-0.0496	-0.0227	0.3628	0.9763	0.8410	-0.2018	0.0520	-0.0040
7 YOE	0.4195	0.9788	0.8555	0.4512	0.9705	0.8559	0.0317	-0.0084	0.0004	0.3515	0.9782	0.8400	-0.0680	-0.0006	-0.0155
9 YOE	0.3084	0.9846	0.8355	0.3832	0.9788	0.8473	0.0748	-0.0058	0.0118	0.2721	0.9878	0.8300	-0.0363	0.0032	-0.0055
18 YOE	0.4512	0.9840	0.8665	0.5011	0.9711	0.8674	0.0499	-0.0129	0.0009	0.3537	0.9885	0.8485	-0.0975	0.0045	-0.0180
31 YOE	0.4649	0.9788	0.8655	0.5193	0.9640	0.8659	0.0544	-0.0148	0.0004	0.3673	0.9865	0.8500	-0.0975	0.0077	-0.0155
**average**	0.4608	0.9672	0.8556	0.5168	0.9479	0.8527	**0.0559**	**-0.0193**	**-0.0028**	0.3636	0.9750	0.8402	-0.0973	0.0078	-0.0154
std. dev.	0.0830	0.0210	0.0115	0.0895	0.0349	0.0145	0.0204	0.0141	0.0095	0.0732	0.0219	0.0112	0.0580	0.0207	0.0085

(YOE = years of experience, sens. = sensitivity, spec. = specificity, accu. = accuracy, std. dev. = standard deviation.

The difference of each metric from that of original images is shown as ‘ -original’.)

**Fig 9:**
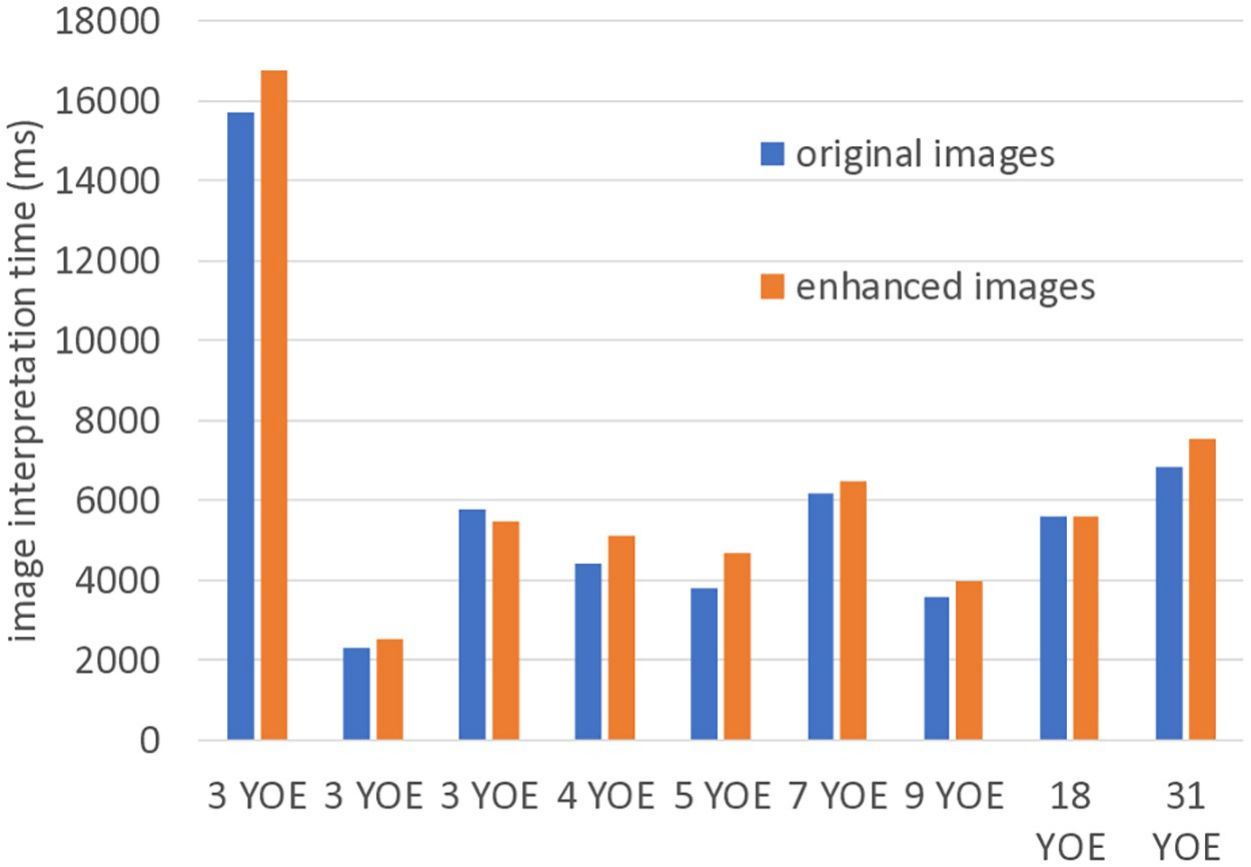
Average image interpretation time of each radiologist. YOE = years of experience.

Fig 9 shows the average reaction times of the radiologists. EGGPALE slightly increased the interpretation time for most radiologists, but the differences were small (the average increase was 444 ms per case).

**Fig 10:**
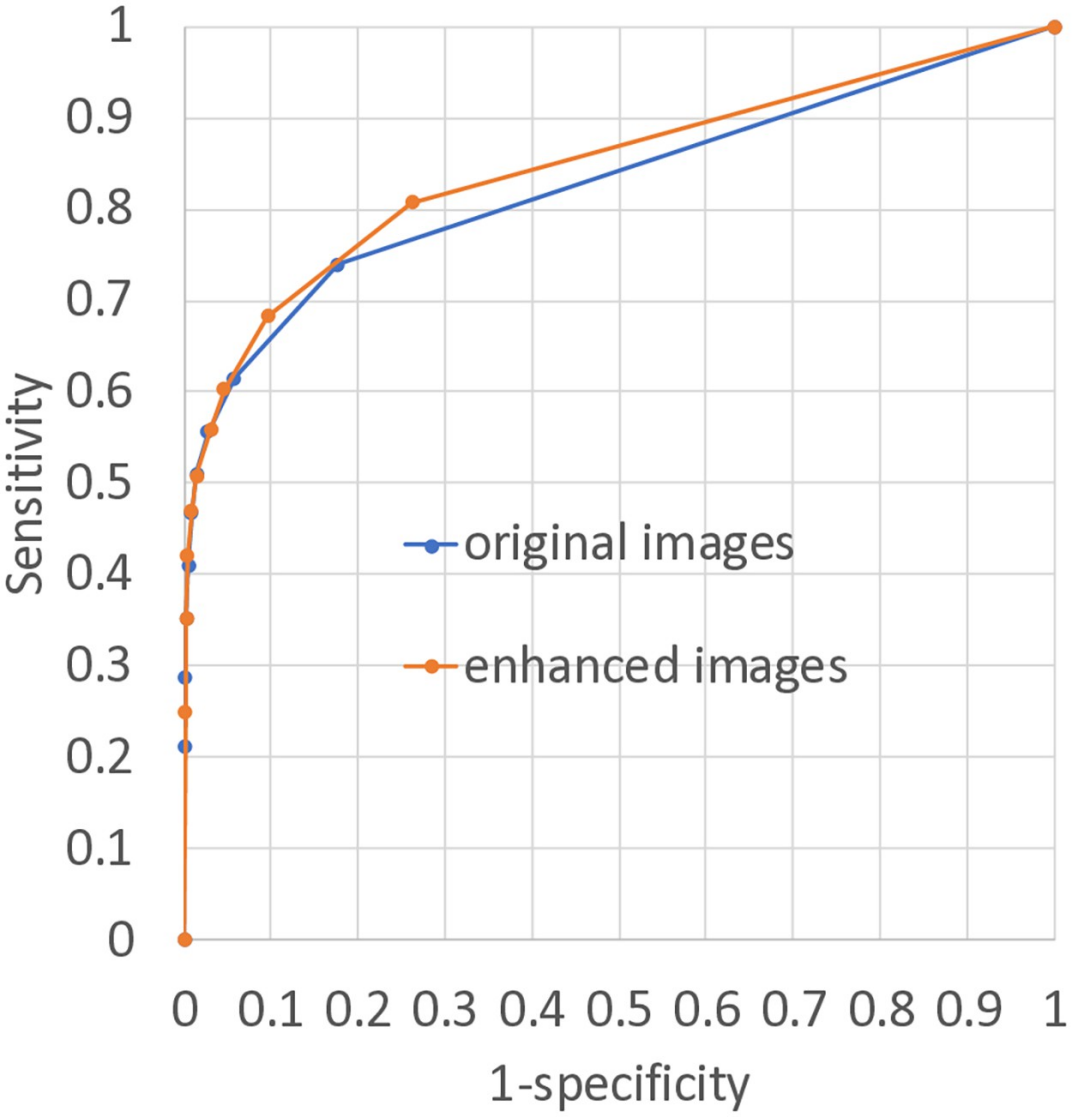
ROC curves of ensemble of all nine radiologists.

Fig 10 shows the ROC curves of the ensemble of all nine radiologists. The areas under the ROC curves (AUCs) were 0.827 and 0.846 without and with EGGPALE, respectively. According to DeLong’s test, the AUCs had a significant difference (*p* = 0.04311).

**Fig 11:**
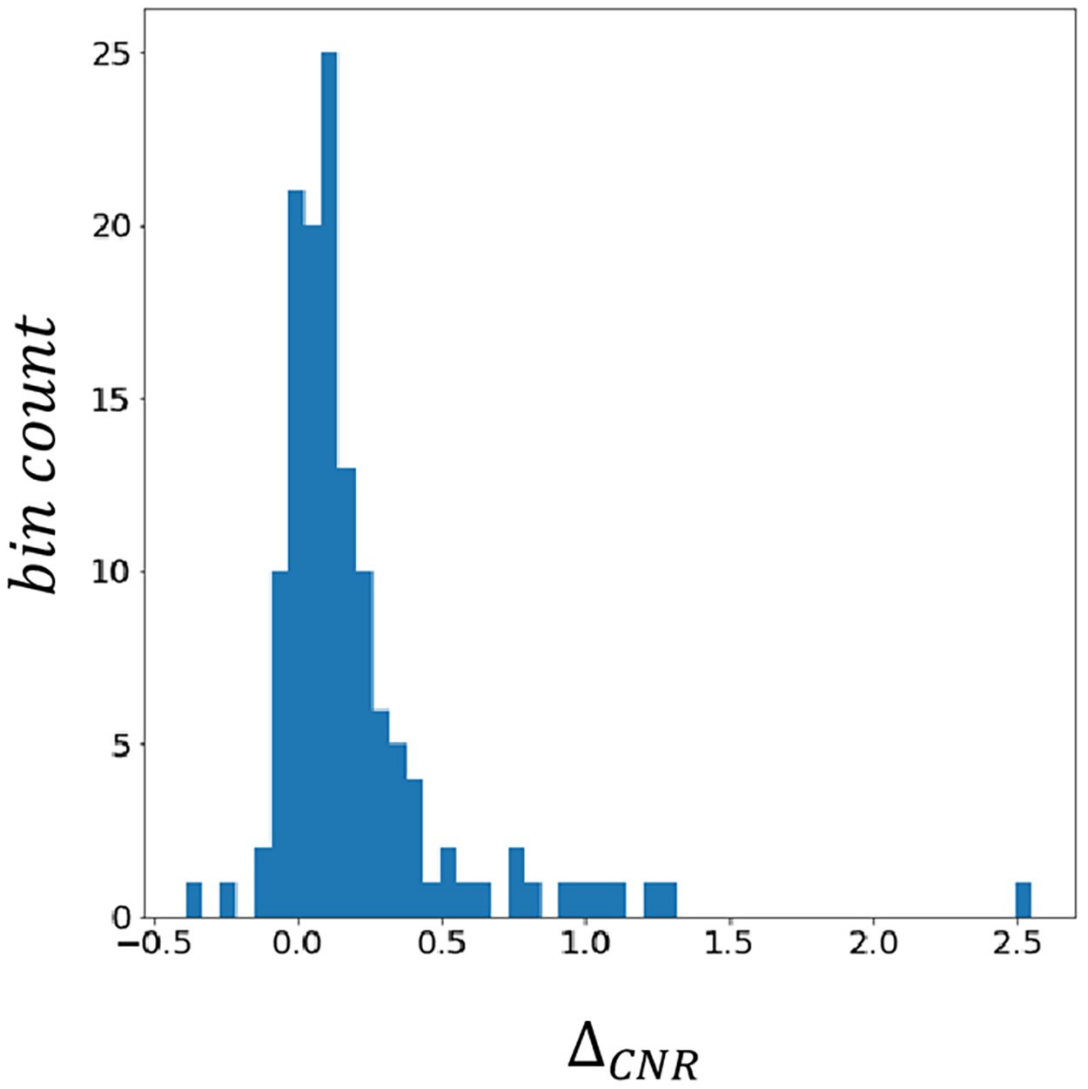
Histogram of Δ_*CNR*_ for 133 nodules in 100 chest X-ray.

Fig 11 shows the histogram of the change in CNR, Δ_*CNR*_, of the nodulous regions. As shown, CNR was improved for most (113 out of 133) of the nodulous regions. Therefore, we concluded that our method can enhance most nodulous regions without significantly changing the contrast of the background (lung field) pixels.

## Discussion

The effective use of CAD software in daily routine work is a difficult problem to address. Sometimes CAD is used with a “concurrent reading” style, in which physicians read the original image and the CAD output simultaneously. Usually, however, CAD is used with a “second reading” style, in which physicians read the original image first and then check the CAD output [43]. Inevitably, both reading styles increase the reading time. This is one of the barriers to the widespread use of CAD software among radiologists, who often read 10^3^–10^4^ images in a single day. In this study, we used the “single reading” style, in which only the CAD output (i.e., enhanced image) was checked. Therefore, the increase in reading time was minimal (444 ms). Although we have not proven that all pathologies can be diagnosed by reading EGGPALE-enhanced chest X-ray images, our experiment proved that, with the enhanced radiographs, radiologists can detect more lung cancers/cancerous lesions with a minimal increase in reading time. We hope that EGGPALE can help and boost the performance of busy radiologists in this sense.

In the image-reading experiment, a statistically significant improvement of sensitivity was observed. It is probable that abnormality enhancement can prevent radiologists from overlooking some tumorous lesions. It is also possible that very faint lesions that are below the recognition threshold of most radiologists became recognizable after EGGPALE processing. Note that distinguishing these two phenomena by experiments is very difficult. However, through our careful subjective observation (Fig 7) and quantitative experiment (Fig 11), it is suggested that lesions below the human recognition threshold can be made visible by EGGPALE. Therefore, it is possible that EGGPALE can not only reduce incidences of overlooked abnormalities but also improve the sensitivity of the X-ray examination itself. Note that the sensitivities in this study were much lower than those of previous studies because we included lesions that were not apparently visible in chest radiographs and only visible in CT.

Along with improved sensitivity, the reduced specificity of all radiologists was observed. The reduction tended to be larger for the radiologists with higher sensitivity. The sensitivity–specificity plot showed that the point for each radiologist on the plot tended to move parallel to the distribution of the nine radiologists’ sensitivities and specificities. In addition, the change in accuracy was approximately zero. Thus, it can be considered that, although EGGPALE changed the threshold of each radiologist on his/her ROC curve, it had little power to push up the ROC curve itself. However, EGGPALE improved the mean sensitivity by 0.0559 and reduced the specificity by 0.0192. Note that the +5.5% of sensitivity improvement was comparable to that (+5.2%) of the commercial chest X-ray nodule CAD (Samsung ALND) reported in [44]. In detail, the average sensitivities of the radiologists without and with their CAD were 65.1% and 70.3%, where the average numbers of false positives per image were 0.2 and 0.18, respectively. Note that their experiment with CAD was performed with a second-reading style (on the other hand, ours is with a single-reading style and is more challenging). In screening tests, the improved sensitivity of chest radiographs can lead to earlier detection of lung cancer patients and thus improve the total outcome of health check programs or medical systems. The deterioration of specificity by 0.0192 would lead to an increase in further CT examinations, but we believe that the use of EGGPALE would be beneficial in health check programs owing to its probable improvement of lung cancer detectability.

The AUC of the ROC of the ensemble of nine radiologists when using EGGPALE was significantly higher than that when they used the original images. In detail, the two curves were mostly identical in the high-specificity, low-sensitivity area, whereas the curve for EGGPALE improved in the low-specificity, high-sensitivity area. This suggests that the proposed method can improve the diagnosis of radiologists, especially when there are two competing diagnoses. In other words, EGGPALE may help radiologists diagnose difficult cases correctly.

For comparison with other nodule enhancement methods, we searched for papers in which nodules are enhanced by (1) contrast enhancement, (2) image filtering, or (3) bone suppression. Although many studies have been reported, as far as we searched, there is no research on (1) or (2) in which the improvement of performance of radiologists was proven in an actual film-reading environment with real X-ray images and nodules. Moreover, we confirmed that no commercially available nodule-enhancing method uses (1) or (2) in the US [45] or EU [46]. Only several works with (3), including [14, 47], proved their effectiveness in performance improvement of radiologists. Therefore, we compared our result with those in these two papers. In [47], operating at a specificity of 90%, sensitivity increased with bone suppressed image from 66% to 71%. In [14], sensitivity was increased from 49.5% to 66.3%, but specificity was decreased from 96.1% to 91.8%. We believe that our result is comparable to theirs. Please note that the experiments in both papers were performed with second reading. Our experiments were performed with single reading only and was therefore more challenging.

The bone suppression system we used for comparison [42] showed poor performance when they were read by radiologists without the original images. In both [14, 47], bone suppression techniques were reported to improve radiologists’ sensitivities when a second reading is performed. However, in our study, a single reading was conducted, that is, the radiologists were blind to the original image. Therefore, now we believe that bone suppression images provide little advantage to radiologists when original images are not available. In other words, a bone suppression image is useful for radiologists if and only if the corresponding original image is also available. Indeed, to the best of our knowledge, all commercially available bone suppression systems are not recommended for use for single reading.

Fig 6 shows that relatively large deformations occur when the existing abnormality itself is large. Generally, the proposed method is designed to change only a relatively small area around an abnormal object if the object is small. In contrast, when the abnormal object is large, a wide or global deformation or a density change is inevitable. Such a deformation or density change severely affects the subtraction image. Note that, in this study, subtraction images are shown for explanatory purpose only and all the film-reading experiments were performed using the emphasized (non-subtracted) images.

This work has some limitations. Our main quantitative experiment was performed only with suspicious lung cancer cases. Chest radiography can be used for a wide range of other important diseases such as tuberculosis, pneumonia, and pneumothorax. Our future work will be to evaluate the diagnostic benefit of EGGPALE for such diseases. Another limitation is that the ROC curve of each radiologist was not available because only a binary input was made for each lung; it was practically too difficult to input multiple confidence levels for all 1,000 cases in our environment. Another future task will be to evaluate datasets with a multiple confidence level system. Finally, our experiment was performed using closed domestic datasets, not open datasets. However, there is no available open and large chest X-ray dataset in which the existence or non-existence of tumor-like lesion was validated by CT examinations.

In summary, the strengths of this study Strengths are: (1) The proposed method exhibits theoretical versatility, allowing for potential application across different scenarios. (2) Experimental validation was conducted using a substantial number of X-ray images and involved the assessment of nine radiologists, enhancing the robustness of the findings. On the other hand, the limitations are: (1) Quantitative evaluation across various pathologies was lacking, potentially limiting the generalizability of the results. However, challenges with regard to computational cost may arise when we train an EGGPALE model on new datasets. (2) The absence of individual ROC curves for each radiologist reduces the granularity of the analysis and may obscure variations in performance. (3) The dataset used was closed and relatively small in size, which could constrain the breadth of insights gained and the applicability of the findings to broader contexts.

## Conclusion

A novel general-purpose abnormality enhancement method, EGGPALE, was presented. It successfully improved the sensitivity of radiologists to cancerous lesions in chest radiographs. Based on the experimental results, the sensitivity demonstrated an average improvement of +0.0559, albeit with an average specificity deterioration of -0.0192. Given the statistically significant improvement in the area under the AUC curve of the ensemble of nine radiologists, we assert the feasibility of our proposed method for lesion enhancement. However, establishing the versatility of the proposed method for various types of lesions remains a focus of our future work. Our future works will also include the application of EGGPALE to other modalities such as mammography and head CT.

## Supporting information

S1 File Appendix. The numerical calculation of *S*.
(DOCX)

S2 File Supplemental material. Semiquantitative image-reading experiment.
(DOCX)
