# Supplementary material for: Deep generative abnormal lesion emphasization validated by nine radiologists and 1000 chest X-rays with lung nodules
Source: PLoS One. 2024 Dec 12;19(12):e0315646. doi: 10.1371/journal.pone.0315646 (PMC11637395; doi:10.1371/journal.pone.0315646)
Supplement: S1 File — The numerical calculation of S. (DOCX) [file pone.0315646.s001.docx]

# Appendix

The numerical calculation of $S$ is performed by QR factorization [47] as follows. First, we compose a $wh\times N$ matrix $\mathbf{Z}^{training}=\left( \begin{matrix} \mathbf{z}^{(1)} & \mathbf{z}^{(2)} & \cdots& \mathbf{z}^{(N)} \end{matrix} \right)$. Then QR factorization of $\mathbf{Z}^{training}$ gives

| $\mathbf{Z}^{training}\boldsymbol{=QR=}\left[ \begin{matrix} \boldsymbol{Q}_{1} & \boldsymbol{Q}_{2} \end{matrix} \right]\left[ \begin{matrix} \boldsymbol{R}_{1} \\ \boldsymbol{0} \end{matrix} \right]\boldsymbol{=}\boldsymbol{Q}_{1}\boldsymbol{R}_{1}\boldsymbol{,}$ | (14) |
| --- | --- |

where $\boldsymbol{Q}$ is a $wh\times wh$ orthogonal matrix, $\boldsymbol{Q}_{1}$ and $\boldsymbol{Q}_{2}$ are matrices with orthogonal columns and sizes of $wh\times N$ and $wh\times(wh-N)$, respectively, and $\boldsymbol{R}_{1}$ is an $N\times N$ upper triangular matrix. Here, each column vector of $\boldsymbol{Q}_{1}$ represents each tangent vector of $S$, and the set of all these tangent vectors forms an orthonormal basis that spans $S$. On the other hand, the column vectors of $\boldsymbol{Q}_{2}$ span the quotient space $\mathbb{R}^{wh}/S$. Therefore, we can represent $S$ as $S=\left\{ \mathbf{z} | \left( \boldsymbol{Q}_{2} \right)^{\mathbf{T}}\boldsymbol{\cdot z=0} \right\}$. This is proven as follows:

| $\boldsymbol{I=}\boldsymbol{Q}^{t}\boldsymbol{Q=}\left[ \begin{matrix} \left( \boldsymbol{Q}_{1} \right)^{T} \\ \left( \boldsymbol{Q}_{2} \right)^{T} \end{matrix} \right]\left[ \begin{matrix} \boldsymbol{Q}_{1} & \boldsymbol{Q}_{2} \end{matrix} \right]\boldsymbol{=}\left[ \begin{matrix} \boldsymbol{Q}_{1}^{T}\boldsymbol{Q}_{1} & \boldsymbol{Q}_{1}^{T}\boldsymbol{Q}_{2} \\ \boldsymbol{Q}_{2}^{T}\boldsymbol{Q}_{1} & \boldsymbol{Q}_{2}^{T}\boldsymbol{Q}_{2} \end{matrix} \right]\boldsymbol{=}\left[ \begin{matrix} \boldsymbol{I} & \boldsymbol{0} \\ \boldsymbol{0} & \boldsymbol{I} \end{matrix} \right]$ | (15) |
| --- | --- |
| $\therefore\left( \boldsymbol{Q}_{2} \right)^{T}\boldsymbol{\cdot}\boldsymbol{Q}_{1}\boldsymbol{=0}$ | (16) |
| $\therefore\left( \boldsymbol{Q}_{2} \right)^{T}\boldsymbol{\cdot}\mathbf{Z}^{training}\boldsymbol{=}\left( \boldsymbol{Q}_{2} \right)^{T}\boldsymbol{\cdot}\boldsymbol{Q}_{1}\boldsymbol{R}_{1}\boldsymbol{=0.}$ | (17) |

Therefore, $\left( \boldsymbol{Q}_{2} \right)^{T}\boldsymbol{\cdot}\mathbf{z}^{\left( i \right)}\boldsymbol{=0, \forall}i$ is satisfied, and thus $S=\left\{ \mathbf{z} | \left( \boldsymbol{Q}_{2} \right)^{T}\boldsymbol{\cdot z=0} \right\}$ includes all $\mathbf{z}^{\left( i \right)}$.

In the abnormality enhancement phase, firstly, a given unseen image $\mathbf{x}^{input}$ is mapped to a point $\mathbf{z}^{input}$ in the latent space. Then, the amplification is performed by moving the point $\mathbf{z}^{input}$ away from $S$. Let the point on $S$ that is closest to $\mathbf{z}^{input}$ be $\boldsymbol{z}_{0}$. Then, $\mathbf{z}_{0}=\boldsymbol{Q}_{1}\left( \boldsymbol{Q}_{1} \right)^{T}\boldsymbol{\cdot}\mathbf{z}^{input}$ is satisfied. Note that multiplying by $\boldsymbol{Q}_{1}\left( \boldsymbol{Q}_{1} \right)^{T}$ can be regarded as a projection operator onto $S$. This is proved as follows. Let $\boldsymbol{z}$ be a certain point on *S*. Therefore, $\left( \boldsymbol{Q}_{2} \right)^{T}\boldsymbol{\cdot z=0}$ is satisfied. Then,

| $\left\vert\boldsymbol{z-}\boldsymbol{z}^{input} \right\vert^{\boldsymbol{2}}$  $\boldsymbol{=}\left\vert\boldsymbol{Q}^{T}\boldsymbol{(z-}\boldsymbol{z}^{input}\boldsymbol{)} \right\vert^{\boldsymbol{2}} \left( \boldsymbol{∵Q}is orthogonal \right)$ $\boldsymbol{=}\left\vert\left[ \begin{matrix} \left( \boldsymbol{Q}_{1} \right)^{T} \\ \left( \boldsymbol{Q}_{2} \right)^{T} \end{matrix} \right]\left( \boldsymbol{z-}\boldsymbol{z}^{input} \right) \right\vert^{\boldsymbol{2}}$ $\boldsymbol{=}\left\vert\left[ \begin{matrix} \left( \boldsymbol{Q}_{1} \right)^{T}\left( \boldsymbol{z-}\boldsymbol{z}^{input} \right) \\ \left( \boldsymbol{Q}_{2} \right)^{T}\left( \boldsymbol{0-}\boldsymbol{z}^{input} \right) \end{matrix} \right] \right\vert^{\boldsymbol{2}}\boldsymbol{(∵}\left( \boldsymbol{Q}_{2} \right)^{T}\boldsymbol{\cdot z=0}\boldsymbol{)}$ $\boldsymbol{=}\left\vert\left( \boldsymbol{Q}_{1} \right)^{T}\left( \boldsymbol{z-}\boldsymbol{z}^{input} \right) \right\vert^{\boldsymbol{2}}\boldsymbol{+}\left\vert\left( \boldsymbol{Q}_{2} \right)^{T}\boldsymbol{z}^{input} \right\vert^{\boldsymbol{2}}$ $\boldsymbol{\geq}\left\vert\left( \boldsymbol{Q}_{2} \right)^{T}\boldsymbol{z}^{input} \right\vert^{\boldsymbol{2}}\boldsymbol{.}$ | (18) |
| --- | --- |

Equality will occur in the final line if and only if $\left( \boldsymbol{Q}_{1} \right)^{T}\left( \boldsymbol{z-}\boldsymbol{z}^{input} \right)\boldsymbol{=0}$. Here, $\boldsymbol{z}_{\boldsymbol{0}}\boldsymbol{=z}$ always satisfies this equation. This is because $\left( \boldsymbol{Q}_{1} \right)^{T}\left( \boldsymbol{z}_{0}\boldsymbol{-}\boldsymbol{z}^{input} \right)\boldsymbol{=}\left( \boldsymbol{Q}_{1} \right)^{T}\left( \boldsymbol{Q}_{1}\left( \boldsymbol{Q}_{1} \right)^{T}\boldsymbol{\cdot}\mathbf{z}^{input}\boldsymbol{-}\boldsymbol{z}^{input} \right)\boldsymbol{=} \left( \boldsymbol{Q}_{1} \right)^{T}\boldsymbol{Q}_{1}\left( \boldsymbol{Q}_{1} \right)^{T}\mathbf{z}^{input}\boldsymbol{-}\left( \boldsymbol{Q}_{1} \right)^{T}\mathbf{z}^{input}\boldsymbol{=I\cdot}\left( \boldsymbol{Q}_{1} \right)^{T}\mathbf{z}^{input}\boldsymbol{-}\left( \boldsymbol{Q}_{1} \right)^{T}\mathbf{z}^{input}\boldsymbol{=0.}$ Therefore, $\boldsymbol{z}=\boldsymbol{z}_{\boldsymbol{0}}$ minimizes $\left| \boldsymbol{z-}\boldsymbol{z}^{input} \right|$.
