# Supplementary material for: Deep generative abnormal lesion emphasization validated by nine radiologists and 1000 chest X-rays with lung nodules
Source: PLoS One. 2024 Dec 12;19(12):e0315646. doi: 10.1371/journal.pone.0315646 (PMC11637395; doi:10.1371/journal.pone.0315646)
Supplement: S2 File — Semiquantitative image-reading experiment. (DOCX) [file pone.0315646.s002.docx]

# Supplemental material

## Experimental settings

We also performed a semiquantitative image-reading experiment with 1,400 images consisting of 100 images $\times$ 14 types of abnormalities included in the ChestX-ray14 dataset. The 14 abnormal findings were atelectasis, cardiomegaly, consolidation, edema, pleural effusion, emphysema, fibrosis, hernia, infiltration, mass, nodule, pleural thickness, pneumonia, and pneumothorax. We randomly extracted 100 images from each of the 14 types of abnormalities using the labels in the ChestX-ray14 dataset. During the random extraction, we excluded images that obviously include no corresponding finding (i.e., obviously mislabeled cases) because the labels provided in the ChestX-ray14 dataset are based on a text mining method and are known to include many errors [48]. Each image was processed by EGGPALE, and the total of 2,800 images (with and without EGGPALE enhancement) were then randomly shuffled. After shuffling, one board-certified radiologist (18 years of experience) checked 2,800 images and determined the clarity of the radiological finding in each image on a scale of 1 to 5. The radiologist was not informed of whether each image was processed by EGGPALE, but he was informed of which abnormal finding was present. After the experiment, the difference between the clarities without and with EGGPALE was statistically tested by a Wilcoxon signed rank test.

## Results

Fig. 12 shows the changes in the clarity of each of the 14 radiological findings before and after EGGPALE enhancement. The improvements in the clarity were statistically significant (p<0.05) for 9 out of the 14 findings (cardiomegaly, consolidation, edema, fibrosis, infiltration, mass, nodule, pneumonia, pneumothorax). On the other hand, the improvements were not significant for 5 findings (atelectasis, emphysema, hernia, effusion, pleural thickness).

Fig. 12. Clarities of 14 types of abnormal findings (evaluated by a radiologist) before and after EGGPALE enhancement. Bold means being statistically significant.


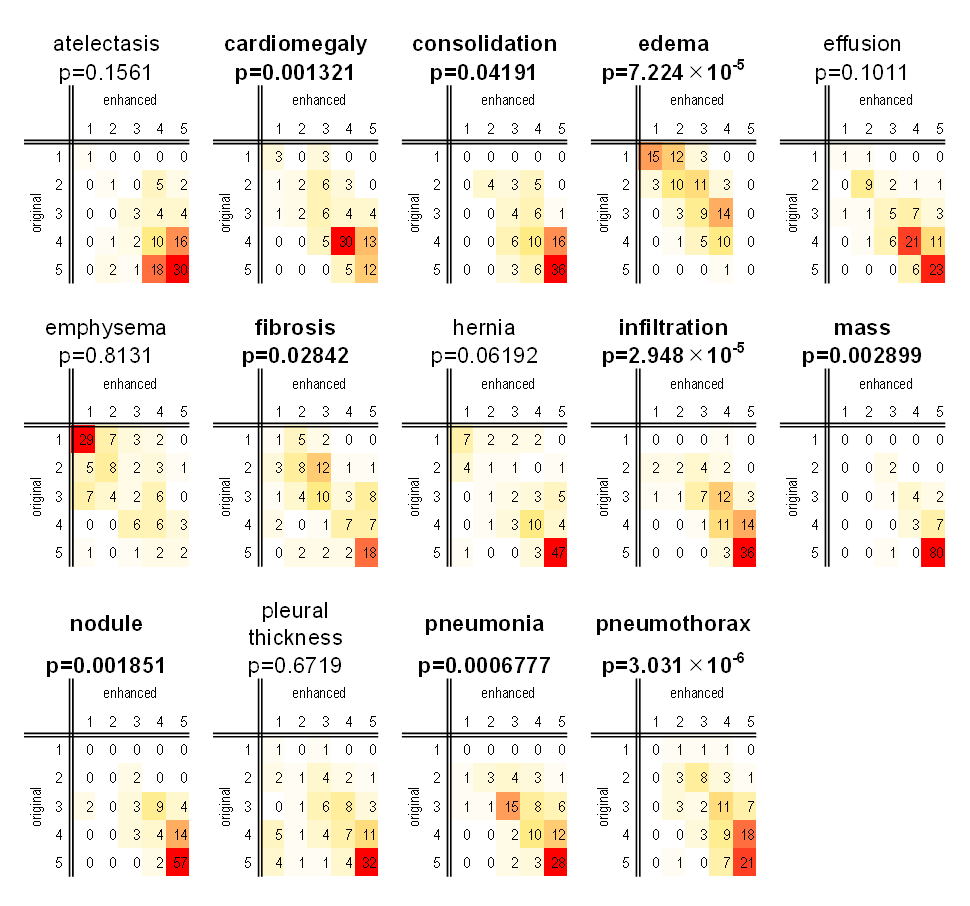


Figure 13 shows the loss curve during the model training. Although the loss descended rapidly and then made a plateau, subjective improvement of the generated images was continually observed until the last epoch.

Fig. 13. Loss curve of Glow in the training phase.

Figure 14 illustrates an example of effect of changing $\beta$ in Eq. 11.

Fig. 14. Example of changing $\beta$.


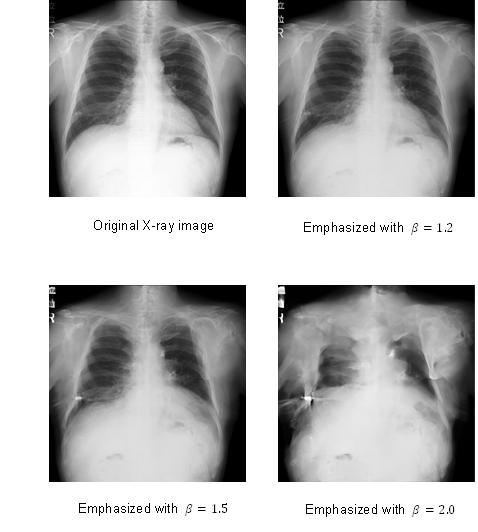


## Discussion

From the results in Fig. 12, we believe that statistically significant differences among the nine diseases indicate the general effectiveness of the proposed emphasization method. In particular, six diseases (cardiomegaly, consolidation, infiltration, mass, nodule, and pneumonia) out of these nine diseases are diseases with mainly local changes. On the other hand, among five diseases without statistically significant differences (hernia, atelectasis, effusion, emphysema, and pleural thickness), two diseases (effusion and emphysema) generally affect the entire lungs. We consider that the reason why the other three diseases (atelectasis, hernia, and pleural thickness) were not statistically significant is that these three diseases were typically too obvious in the original images so that their enhancement has little effect on film reading. Although EGGPALE emphasizes various diseases, it is probably more effective for local lesions than for diffuse lung diseases.
